# Effect of Autologous Conditioned Plasma Injections in Patients With Knee Osteoarthritis

**DOI:** 10.1177/23259671231184848

**Published:** 2023-07-28

**Authors:** Jasmijn V. Korpershoek, Lucienne A. Vonk, Giuseppe Filardo, Esmee C. Kester, Nienke van Egmond, Daniël B.F. Saris, Roel J.H. Custers

**Affiliations:** * Department of Orthopaedics, University Medical Center Utrecht, Utrecht, the Netherlands.; † Service of Orthopaedics and Traumatology, Department of Surgery, EOC, Lugano, Switzerland.; ‡ Faculty of Biomedical Sciences, Università della Svizzera Italiana, Lugano, Switzerland.; § Department of Orthopedic Surgery and Sports Medicine, Mayo Clinic, Rochester, Minnesota, USA.; ∥ Department of Reconstructive Medicine, University of Twente, Enschede, the Netherlands.; *Investigation performed at Department of Orthopaedics, University Medical Center Utrecht, Utrecth, The Netherlands.*

**Keywords:** ACP, PRP, knee osteoarthritis, injections, platelets

## Abstract

**Background:**

Autologous conditioned plasma (ACP) is a commercially available platelet concentrate with promising results from clinical trials.

**Purpose:**

To evaluate the clinical outcome after 3 consecutive injections of ACP in patients with knee osteoarthritis (OA) and study the influence of ACP composition and different patient factors as predictors of treatment effect.

**Study Design:**

Case series; Level of evidence, 4.

**Methods:**

This prospective case series included 260 patients (307 knees) who received ACP treatment for knee OA. The mean patient age was 51 ± 10 years. Improvement up to 12 months’ follow-up was measured using the Knee injury and Osteoarthritis Outcome Score (KOOS). ACP composition was analyzed in 100 patients. The predictive value of age, sex, history of knee trauma, Kellgren-Lawrence OA grade, body mass index, and ACP composition was evaluated using generalized estimating equations.

**Results:**

The mean overall KOOS improved from 38 ± 14 at baseline to 45 ± 18 at 3 months, 45 ± 18 at 6 months, and 43 ± 18 at 12 months (all *P* < .05); 40% of patients achieved an improvement above the minimal clinically important difference (MCID) of 8 after 6 months and 33% after 12 months. The variation in ACP composition did not correlate with KOOS (*P* > .05). Older age led to a greater clinical benefit (β = 0.27; *P* = .05), whereas bilateral treatment predicted worse outcomes (β = –5.6; *P* < .05).

**Conclusion:**

The improvement in KOOS after treatment with ACP did not reach the MCID in most study patients. Older age was a predictor for better outcomes. The composition of ACP varied between patients but did not predict outcomes within the evaluated range. The study findings show the limited benefit of ACP treatment for knee OA and call for caution with routine use in clinical practice.

Worldwide, knee osteoarthritis (OA) affects 5% of the population.^12^ There is a need for knee-preserving treatments aimed at reducing symptoms, regenerating the damaged tissues, or at least restoring joint homeostasis and slowing OA progression. Platelet-rich plasma (PRP) is a concentrate of autologous platelets that has emerged as a treatment for OA because of its high concentrations of growth factors and cytokines.^7,17,18^ Although the first applications of PRP for knee OA reached the clinic 2 decades ago, the efficacy of this treatment is still the subject of debate. Randomized controlled trials (RCTs) showed results varying from strong effects on pain and symptoms^29^ to no beneficial effect compared with viscosupplementation^10^ or placebo.^4^ The composition of PRP differs between different preparation methods or manufacturers,^7,17,18^ and inter- and intrapatient variability exist because of variation in the composition of whole blood.^5^ Autologous conditioned plasma (ACP; Arthrex), a commercially available single-spin leucocyte-poor PRP, is widely used in clinical practice for the treatment of knee OA, even though it is often not reimbursed by insurance.^8^ In a previous case series,^16^ injections with ACP did not lead to a clinically relevant improvement in patient-reported outcome measures (PROMs). To date, we know of no studies with large-scale product composition assessments.

The purpose of this study was to evaluate the effect of ACP for the treatment of knee OA in patients referred to a tertiary center from data gathered using a prospective registry. We hypothesized that patient characteristics such as age and body mass index (BMI), as well as higher platelet concentrations in ACP aliquots, would predict better treatment outcomes and could be used to optimize PRP treatment for OA.

## Methods

### Study Design

This prospective case study included patients treated with ACP at a single institution between March 2017 and April 2020. The study was conducted according to the guidelines of the World Medical Association’s Declaration of Helsinki, and we followed the Strengthening the Reporting of Observational Studies in Epidemiology guidelines for reporting cohort studies.^26^ Ethics committee approval was received for the study protocol, and all patients provided written informed consent for participation in the study. Patients included in the analysis of ACP composition provided additional consent for analysis of their material.

### Participants

Patients who had symptomatic multicompartmental knee OA in all radiographic stages were offered ACP treatment free of charge. To select a representative patient population for our clinic, we included all patients who underwent ACP injections at our center and had at least 12 months of follow-up data if they met the following criteria: chronic symptomatic OA (>6 months), no concomitant treatment (such as bracing, injections, or surgical treatment; patients undergoing physical therapy were not excluded), and sufficient understanding of the Dutch language. Exclusion criteria were prior treatment with ACP, <3 ACP injections received (this was part of our study protocol), and inflammatory joint disease or other generalized musculoskeletal pathologies affecting the knee.

### Data Collection

Age, sex, history of knee trauma (meniscus injury, anterior cruciate ligament rupture, cartilage defect, tibia plateau fracture), and BMI were collected from the electronic patient database for all patients. No differentiation was made between treated or untreated knee trauma. Data on comorbidities were not collected in this knee registry, except for inflammatory conditions that were a contraindication for participation.

### Kellgren-Lawrence OA Grading

Weightbearing anterior-posterior knee radiographs were taken of all patients before undergoing treatment. Kellgren-Lawrence grades were determined by 3 reviewers (2 orthopaedic surgeons with over 10 years of experience [N.v.E. R.J.H.C.] and 1 clinical researcher [J.V.K]). If the difference in grades was 2 or more between 2 reviewers, consensus was reached in a meeting. If 1 reviewer valued the Kellgren-Lawrence either 1 grade lower or higher than the other 2 reviewers, the value that was agreed on by 2 reviewers was used. The interrater reliability was estimated by the intraclass correlation coefficient using a 2-way mixed model with absolute agreement. There was excellent agreement between reviewers, with a Cronbach α of 0.93 (internal consistency of 0.7-0.9 is considered good agreement, 0.6-0.7 acceptable, and 0.5-0.6 poor).

### ACP Administration

ACP was prepared according to the recommendations for the Arthrex ACP double-syringe system: 15 mL autologous peripheral blood was drawn and centrifuged at 360*g* for 5 minutes at room temperature (Hettich Centrifuge). This resulted in a variable volume of ACP in the top layer that was aspirated into the inner syringe of the double-syringe system; the bottom layer with erythrocytes was discarded. The ACP was immediately injected into the knee joint with an aseptic technique using a superolateral approach with the patient in the supine position without ultrasound guidance. This technique has an accuracy of 91%.^13^ No platelet activation was performed. Time between blood draw and injection was a maximum of 30 minutes, during which the product was kept at room temperature. Three intra-articular injections were provided in the outpatient clinic with a 1-week interval. Of 100 patients who provided additional consent for ACP analysis, approximately 200 µL residual ACP of all 3 ACP products was stored for 2 to 3 hours at room temperature in an Eppendorf tube with 30 µL citrate to prevent clotting and used for composition analysis. ACP composition was analyzed using a CELL-DYN Emerald hematology analyzer (Abbott BV). Platelet, erythrocyte, and leucocyte concentrations were measured in duplicate. The volume of injected ACP was documented at each time of injection. Analyses of whole blood were not performed; therefore, no platelet recovery rates could be calculated. No rehabilitation protocol or immobilization was advised, and patients were allowed to undergo physical therapy of their preference. No active screening for untoward effects was performed.

### Patient-Reported Outcome Measures

PROMs were collected before treatment (baseline) and at 3, 6, and 12 months after treatment using an online survey tool (OnlinePROMS; Interactive Studios). Questionnaires included the Knee injury and Osteoarthritis Outcome Score (KOOS), EuroQol 5 Dimensions 5 levels (EQ5D-5L), and numeric rating scale for pain (NRS-pain) during activity and in rest. Scores ranged from 0 (worst) to 100 (best) for KOOS and EQ5D-5L and from 0 (best) to 10 (worst) for NRS-pain. Patients filled in separate questionnaires for bilateral treatment. For patients who underwent other treatment before completing follow-up, the last values were carried forward to address selective loss to follow-up.

### Data Analysis and Statistics

Data were analyzed using Statistical Package for the Social Sciences (SPSS) (Version 26.0.0.1; IBM). Baseline characteristics were reported as means and standard deviations or as number of patients and percentage of total. Outcomes were reported as mean with 95% confidence interval. Missing data were not imputed except for patients who underwent alternative treatment. With generalized estimating equations (GEEs), all available data of all patients are included in the model. *P* values <.05 were considered statistically significant.

The improvement in the mean score on the 5 KOOS subscales of Pain, Symptoms, Activities of Daily Living, Sport and Recreation, and Knee-related Quality of Life (KOOS_5_) was assessed using GEEs with multiple measurements, taking into account the possible correlation of bilaterally treated patients. Change in KOOS_5_ was compared with the minimal clinically important difference (MCID) for KOOS. The authors of the KOOS recommend using a score of 8 for the MCID, and the MCID for the different subscores ranged between 8.2 and 12.5 in a PRP cohort study.^6,22^ For NRS-pain, the scores for pain in rest and activity were analyzed separately.

The independent predictive values of sex, history of knee trauma (meniscus injury, anterior cruciate ligament rupture, cartilage defect, tibial plateau fracture), Kellgren-Lawrence grade, BMI, and composition of ACP for KOOS were analyzed using GEEs, correcting for follow-up duration and age as fixed effects. A linear model with an unstructured correlation matrix and identity link function was used. Significant predictive value was defined as *P* < .05 for the variable. The factors were assessed as main effects. Collinearity was assessed using a correlation matrix and linearity with a scatterplot. For the Kellgren-Lawrence grade, repeated contrast coding was used to compare grade 0 to grade 1, grade 1 to grade 2, and so on. To assess the influence of loss to follow-up, characteristics of patients who completed the 12-month survey were compared with patients who did not complete the follow-up. Intrapatient correlations among ACP compositions were assessed using the Pearson correlations.

## Results

### Baseline Characteristics and ACP Composition

**Figure 1.**
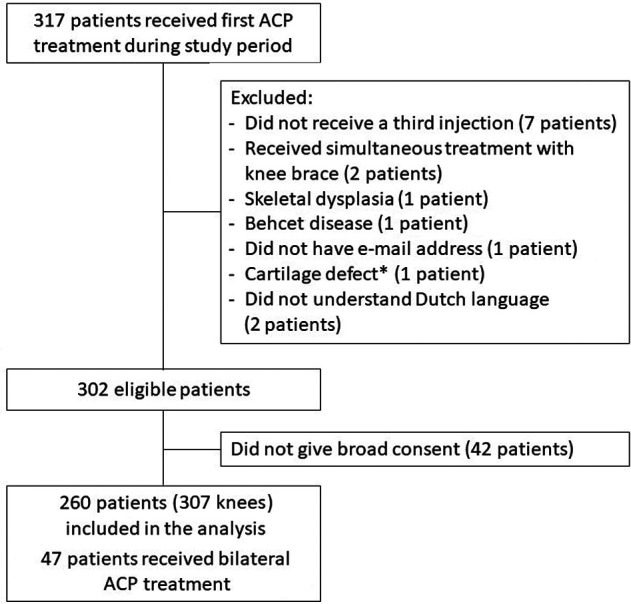
Flowchart of patient inclusion. *^a^* The patient with a cartilage defect was excluded, as this was a traumatic defect with no degenerative disease involved.

Overall, 260 patients (307 knees) were included in the analysis. An overview of patient inclusion can be found in Figure 1. In 80 knees, ACP injections were administered with an interval of more than 1 week because of scheduling issues. Baseline characteristics of the study patients are shown in Table 1. Baseline patient characteristics were complete except for BMI (24% missing). A total of 17 knees underwent alternative treatment before completing the follow-up of 12 months. Alternative treatments were knee joint distraction (6 knees), ligament surgery (1 knee), removal of a loose body (1 knee), genicular nerve block (1 knee), treatment at another clinic (2 knees), knee brace (1 knee), total knee replacement (2 knees), new series of ACP (2 knees), and a program provided by rehabilitation medicine (1 knee). Response rates for the PROMs were 89% at baseline, 88% at 3 months, 80% at 6 months, and 74% at 12 months.

**Table 1:** Baseline Characteristics of the Included Patients (N = 260 Patients, 307 Knees)*^a^*

Characteristic	Value
Age, y	51 ± 10
Female sex	183 (59)
BMI,*^b^* kg/m^2^	29 ± 5
History of knee trauma	132 (43)
KOOS_5_	37 ± 13
KOOS subscales	
Sport and Recreation	13 ± 16
Pain	43 ± 16
Symptoms	51 ± 18
Activities of Daily Living	52 ± 18
Knee-related Quality of Life	24 ± 15
NRS, pain at rest	5.0 ± 2.3
NRS, pain during activity	7.1 ± 1.8
EQ5D-5L	65 ± 19
ACP treatment in contralateral knee	94 (31)
Kellgren-Lawrence grade	
0*^c^*	9 (2.9)
1	59 (19)
2	107 (35)
3	96 (31)
4	36 (12)

*^a^* Data are presented as mean ± SD or number (%). ACP, autologous conditioned plasma; BMI, body mass index; EQ5D-5L, EuroQol 5 Dimensions 5 levels; KOOS, Knee injury and Osteoarthritis Outcome Score; KOOS_5_, the average score on the 5 KOOS subscales; NRS, numeric rating scale.

*^b^* BMI missing for 74 knees (24%).

*^c^* These patients had magnetic resonance imaging or arthroscopically diagnosed osteoarthritis.

**Figure 2.**
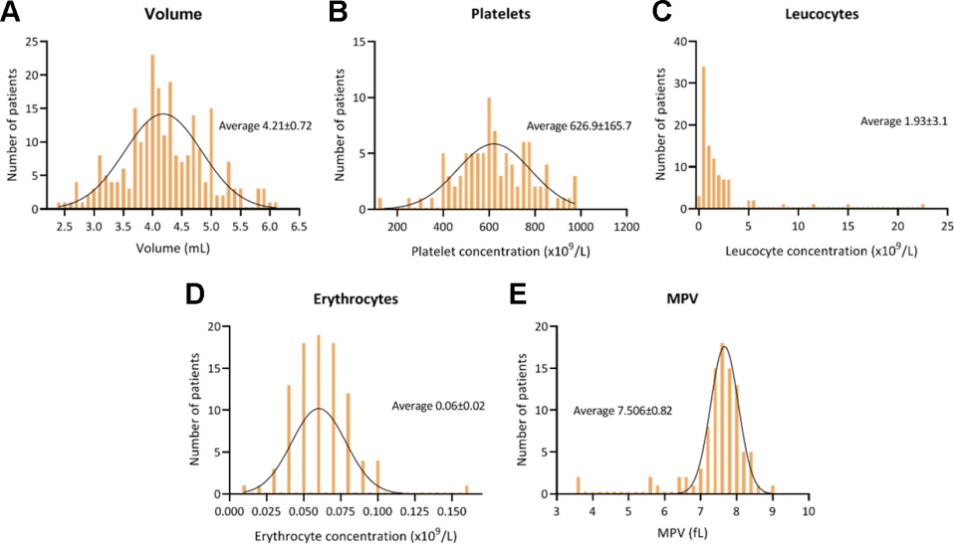
Mean and distribution of ACP characteristics in 100 patients. MPV, mean platelet volume.

The mean injected ACP volume and platelet concentration, leucocyte concentration, erythrocyte concentration, and mean platelet volume (MPV) were variable among patients (Figure 2). Based on the estimated population platelet concentration in whole blood,^5^ the estimated concentration was 2 to 3 times, with a platelet recovery of 70%. Platelet concentration, leucocyte concentration, erythrocyte concentration, and MPV of the 3 consecutive injections that patients received were not significantly correlated, although intrapatient variation was smaller than interpatient variation. For example, the mean of the standard deviations of the platelet concentration of 3 injections in 1 patient was 162, whereas the standard deviation between all measurements in the population was 215. Injected volumes of the consecutive injections were correlated with a mean Pearson correlation of 0.6. Characterization of the ACP according to Kon et al^15^ can be found in Appendix Tables A1 and A2.

### Effectiveness of ACP

**Figure 3.**
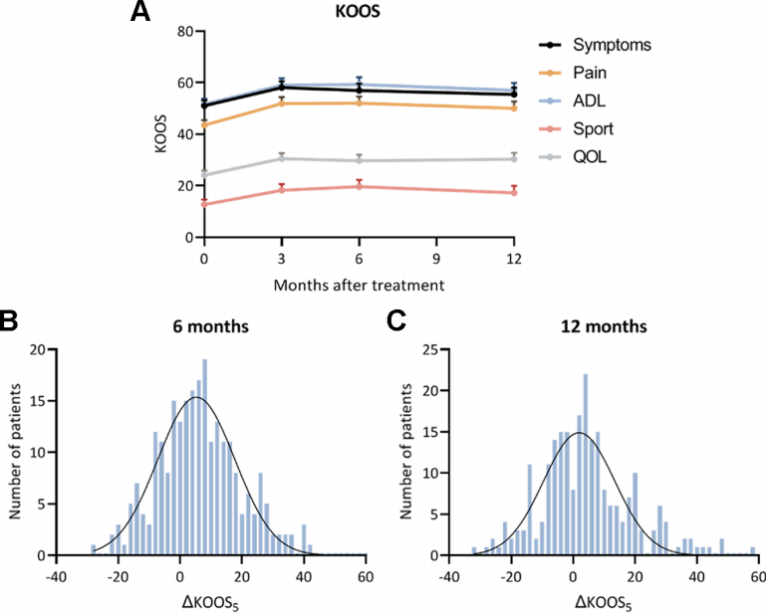
(A) Mean Knee injury and Osteoarthritis Outcome Score (KOOS) values at baseline and after autologous conditioned plasma treatment. Error bars indicate 95% CIs. (B, C) Distribution in the study population of the change in KOOS_5_ at (B) 6 months and (C) 12 months after treatment. ADL, Activities of Daily Living; ΔKOOS_5_, the change in average score on the 5 KOOS subscales; QOL, Knee-related Quality of Life.

The KOOS_5_ improved significantly from baseline to all time points (*P* < .05). The improvements were comparable in all subscales (Table 2 and Figure 3). Of the available data, an improvement above the MCID was achieved in 45% of patients at 3 months, 40% of patients at 6 months, and 33% of patients at 12 months. The change in KOOS from baseline was normally distributed around the mean improvement.

**Table 2:** Outcomes After ACP Injections*^a^*

Characteristic	Baseline	3 Months	6 Months	12 Months
KOOS_5_	38 (36-40)	45 (42-48)	45 (42-48)	43 (40-45)
NRS-pain in rest	5.0 (4.7-5.3)	3.9 (3.5-4.3)	3.8 (3.5-4.2)	4.0 (3.6-4.4)
NRS-pain in activity	7.0 (6.8-7.3)	6.1 (5.7-6.4)	6.2 (5.9-6.5)	6.2 (5.9-6.6)
EQ5D-5L	66 (64-69)	66 (64-69)	67 (65-70)	67 (64-70)

*^a^* Data are presented as mean (95% CI). EQ5D-5L, EuroQol 5 Dimensions 5 levels; KOOS, Knee injury and Osteoarthritis Outcome Score; KOOS_5_, the average score on the 5 KOOS subscales; NRS, numeric rating scale.

The patients who did not complete the 12-month follow-up (for a reason other than they received alternative treatment) did not deviate from the available KOOS_5_ scores. These patients did have a BMI of 1.6 points higher than the patients who completed follow-up (*P* = .03), but the other baseline characteristics were similar between the groups. NRS-pain in rest and in activity improved from baseline to all time points (*P* < .05). EQ5D-5L did not change significantly.

Among the evaluated factors, older patients had increased improvement, as expressed by a coefficient of 0.27 (95% CI, 0.01-0.54), and an increase of 1 year in age predicted a 0.27-point improvement in KOOS between each assessment (ie, baseline versus 3 months, 3 versus 6 months, 6 versus 12 months). Lack of history of knee trauma, lower injected volume, and lower BMI were significant predictors in the univariate analysis, but they were correlated with age and lost significance after correction for age in the multifactorial model. Bilateral treatment was a predictor for poorer clinical outcome (coefficient, –5.6; 95% CI, –11 to 0.2; *P* = .04). Patients with Kellgren-Lawrence grade 1 responded worse to treatment than patients with Kellgren-Lawrence grade 0 who had magnetic resonance imaging or surgical evidence of OA (coefficient, –11.6; 95% CI, –21.0 to –2.2; *P* = .02). No other factors significantly predicted outcomes in this series (Table 3).

**Table 3:** Analysis of Patient and ACP Factors in the Prediction of KOOS_5_ Using a Generalized Estimating Equations Model*^a^*

Factor	Coefficient (95% CI)	*P*
History of knee trauma	0.0 (–8.5 to 8.5)	>.99
Male sex	5.1 (–9.4 to 20)	.49
Bilateral treatment	–5.6 (–11 to –0.2)	**.04**
Kellgren-Lawrence grade*^b^*		
1 (vs 0)	–11.6 (–21.0 to –2.2)	**.02**
2 (vs 1)	–5.2 (–12.1 to 1.7)	.14
3 (vs 2)	0.0 (–8.7 to 8.7)	>.99
4 (vs 3)	–0.5 (–6.6 to 5.6)	.87
BMI	0.1 (–0.3 to 0.6)	.56
Mean platelet concentration, ×10^9^/L	0.0 (0.0 to 0.0)	.79
Mean leucocyte concentration, ×10^9^/L	–0.7 (–1.7 to 0.3)	.17
Mean erythrocyte concentration, ×10^9^/L	–236.5 (–427 to 1.1)	.06
Mean injected volume, mL	–3.8 (–8.2 to 0.5)	.09
Mean platelet volume, fL	–3.7 (–7.8 to 0.3)	.08

*^a^* Corrected for age and months after treatment. Boldface *P* values indicate statistical significance (*P* < .05). BMI, body mass index.

*^b^* Each Kellgren-Lawrence grade was compared with the grade just before it using repeated-contrast coding.

Of all included patients, 50 patients (60 knees) returned for a second series of 3 ACP injections. At baseline, there were no differences between patients who returned for a second series and those who did not return. At 6 months, patients who later underwent a second series of ACP had a ΔKOOS_5_ of 12 (95% CI, 7-18), whereas other patients had a ΔKOOS_5_ of 6 (95% CI, 4-8). At 12 months, patients who later returned for a second series of ACP had a ΔKOOS_5_ of 7 (95% CI, 2-10), and other patients had a ΔKOOS_5_ of 4 (95% CI, 2-7). None of the patients developed a joint infection after injection. Other adverse effects were not actively registered.

## Discussion

In most patients in this study, intra-articular injections with ACP did not result in an improvement of KOOS above the MCID. The limited number of patients who reported a clinically relevant improvement in the current study (33% at 1 year) is also illustrated by the small number of patients who returned for a second series of treatment (50 patients [60 knees]). The ACP composition varied between patients, but this variation did not predict clinical outcomes. Older age and unilateral treatment predicted better outcomes of ACP treatment for knee OA and could be used to improve treatment indications and outcomes.

The low clinical benefit of ACP injections for the treatment of knee OA is in agreement with our previous report^16^ and a recently published RCT comparing PRP with placebo injections.^4^ Both studies used a low concentration of platelets, with the platelet dosage administered being within the low range of blood-derived treatments considered within PRP products in the field. While it is possible that higher doses could lead to different outcomes, a wide range of results has been reported also by previous reports on the same product. ACP was previously investigated by other authors for the treatment of knee OA, with heterogeneous findings. Using the same evaluation tool, Cerza et al^9^ and Smith^24^ reported highly satisfactory results at 6 months, with a total Western Ontario and McMaster Universities Osteoarthritis Index (WOMAC) score improvement of 43 in 60 patients (67 years old) and 36 points in 15 patients (54 years old), respectively, while recently, Sun et al^25^ documented with the same tool at the same follow-up a much lower score improvement of 15 points in 38 patients (58 years old) treated with ACP for knee OA. In this light, the larger series documented in our study adds important information to the debate on ACP effectiveness. The low percentage of patients reaching an MCID warrants caution in offering this treatment solution to patients, who should be aware of the results and have proper expectations. In addition, the wide range of results in the literature in different settings with different patient populations could be explained by various factors that should be identified to aim at understanding the real potential and indications for this biological treatment approach.

This study aimed to improve treatment indications and clinical outcomes after ACP treatment by identifying patient or ACP factors that predict better outcomes. Although patients without radiographic OA (Kellgren-Lawrence grade 0) had higher improvements, the small numbers of patients with Kellgren-Lawrence grade 0 limit the generalizability of our findings. None of the other Kellgren-Lawrence grades predicted outcomes, like in a recent large RCT.^4^ BMI was not predictive of worse outcomes. However, the BMI of patients who did not fill in the 12-month surveys was higher at baseline than the BMI of the other patients, suggesting selective loss to follow-up. In our cohort, older age and unilateral treatment predicted better outcomes. Function is worse in patients with bilateral complaints^27^ and influences PROMs.^21^ Age is a controversial factor; patient age did not affect outcomes in a retrospective cohort study focused on identifying predictors of effectiveness of PRP therapy for knee OA,^23^ while other authors showed that older age increased the odds for treatment failure.^1^ However, the mean patient age in these studies was 60 to 70 years, whereas the mean age in our cohort was 51 years. Thus, based on these and our findings, optimal patient age could lie between 50 and 70 years. The age of the patients representing our cohort is relatively young compared with other cohorts and might be attributed to the fact that the patients were included in a tertiary referral center. The high incidence of posttraumatic OA and low subscales for sports and recreation could indicate that our patient population was previously active and experienced major limitations in sports activities. While most of the literature reported good results in older patients (mean age, 49-65 years),^11^ poorer results have been reported for younger (mean age, 41 years) and active patients seeking a return to sport,^2^ with a more disappointing outcome in line with the findings of this study. Active patients affected by knee OA have less satisfactory results since only half can achieve the same sport level as before the onset of symptoms,^2^ and they should be made aware of their low chances of benefit from this treatment. The activities that patients wish to perform should be considered in future studies. Similar to our findings, in an RCT of PRP treatment in a sports clinic, patients around 50 years of age with around a 50% incidence of posttraumatic OA were included,^19^ and comparable improvements were reported in the PRP arm of the study. In the current study, BMI, history of knee trauma, and volume of injected ACP were significant independent predictors but correlated with age and lost significance after correction for age. Interestingly, older age correlated with a higher yield in ACP volume. Although it is known that platelet numbers decline with aging,^14^ the relation between ACP volume and age is unclear. These results suggest that age is the most important factor for predicting outcomes after ACP treatment in clinical practice.

When assessing the correlation of ACP composition to treatment outcomes, we found that, similar to earlier reports,^15,28^ platelet concentration did not correlate with treatment outcomes. The naturally occurring variation in ACP composition did not predict clinical outcomes. The MPV correlates to the content of platelets,^15^ and thus, there could be an effect of MPV on clinical outcomes. However, the small variations in MPV did not predict outcomes in our study. In a study by Bansal et al,^3^ injection of a single dose of 10 billion platelets resulted in reasonable improvements in the WOMAC score. The absolute number of platelets per injection was on average 4 times lower in our study. Platelet numbers in ACP could be too low, and these results might favor administration of a single high dose of platelets instead of 3 injections with lower concentrations. As long as no direct dosing studies are performed, firm conclusions on optimal composition cannot be drawn due to the heterogeneity in the studied products and patient populations. Dosing studies on platelet concentration, leucocyte concentration, and MPV could lead to a more bioactive PRP, but large patient numbers would need to be enrolled in such studies to sort out all of the contributing factors.

In a study by Zahir et al,^28^ an in vitro inflammatory coculture model was established to predict which patients benefit more from treatment, as BMI, age, sex, and platelet concentration did not predict outcomes. Patients who did not respond well to PRP treatment also lacked the inhibition of proinflammatory cytokine tumor necrosis factor–α in the in vitro model. Thus, certain factors in PRP resulted in differences in bioactivity among patients, but at this time, it is unclear which factors are responsible. Identifying these factors could improve patient selection or development of a synthetic or an allogeneic PRP with a higher bioactivity.

### Limitations

This study is not without limitations. This was a noncontrolled study; therefore, the size of a placebo effect in the current study is unclear. The placebo effect is likely considerable, as injections with saline lead to an improvement exceeding the MCID in half of the patients,^20^ similar to our findings with ACP injections. Furthermore, this study does not compare with other alternatives such as steroid and hyaluronic acid, as these are not provided routinely in our clinic. Moreover, the effect of regression to the mean in this study is unclear but likely considerable due to the characteristics of the study group. In addition, patients were to undergo ACP treatment using broad inclusion criteria. Therefore, this study contains OA in all grades and etiologies. Last, we did not control for intake of analgesics or physical therapy. However, this study shows the results of ACP treatment in a real-world setting, and the large number of patients evaluated in this series and broad inclusion and exclusion criteria are strengths for generalizability of the findings. Furthermore, the large number of patients allowed us to identify factors that predict better outcomes and could optimize patient selection. However, we did not gather data on comorbidities, smoking status, and so on. Adding these factors to the registry could lead to important insights. Another limitation of this study is that we did not analyze whole-blood samples, and we cannot relate the ACP composition with these values. Therefore, we do not have any information of the variations in the ACP preparation process between patients or injections. The analysis of the predictive value for ACP composition is a strength of this study.

## Conclusion

Intra-articular injections with ACP do not lead to a clinically relevant improvement in most patients with knee OA, and therefore, ACP should not be used as a routine treatment in the clinical setting. Instead, patients who might benefit more from treatment should be selected. This study identified older age as an important predictor for a better outcome. As the patients in the current cohort were relatively young, the optimal age for ACP treatment might lie between 50 and 70 years. Other predicting patient factors should be identified. The naturally occurring variation in blood derivatives composition did not predict clinical outcomes in this series treated with ACP, and optimizing the composition of PRP products should be the focus in follow-up research, preferably using well-designed dosing studies.

## Appendix

**Table A1:** Characterization of the ACP According to Kon et al^15^ *^a^*

Number	Characteristic	Digit of ACP
Number 1 (N_1_)	Basal platelet concentration in blood	Not measured
Number 2 (N_2_)	Platelet concentration in PRP	4 (400,000-500,000)
Number 3 (N_3_)	Red blood cells in PRP	1 (>1 × 10^6^)
Number 4 (N_4_)	White blood cells in PRP	0 (less than baseline)
Number 5 (N_5_)	External activation	0 (endogenous activation)
Number 6 (N_6_)	Calcium addition	0 (no)

*^a^* PRP, platelet-rich plasma.

**Table A2:** Summary of characteristics for in vitro PRP studies according to Kon et al^15^ *^a^*

1. PRP preparation	
Initial blood volume	15 mL
Anticoagulant	None
System	Close
Centrifugation	Single centrifugation at 360*g* for 5 min
Final PRP volume	4.2 ± 0.72 mL (also see Figure 2)
2. PRP characteristics	
PRP type (N_1_ N_2_ – N_3_ N_4_ – N_5_ N_6_)	Not measured: 4 – 10 – 00
MPV	7.5 fL
Red blood cells	0.06 × 10^9^/L
White blood cells	1.9 × 10^9^/L
Neutrophils	Not measured
Lymphocytes	48%
Monocytes	Not measured
Eosinophils	Not measured
Basophils	10%
Activation	None
Middle-sized cells*^b^*	7%
3. Application characteristics	
Formulation type	Liquid
Administration route	Superolateral injection without image guidance
Dosage	3 injections with 1-week intervals
Volume	4.2 ± 0.72 mL (also see Figure 2)
Tissue	Knee
Pathology	Osteoarthritis

*^a^* MPV, mean platelet volume; PRP, platelet-rich plasma.

*^b^* Leucocytes not classified as lymphocytes or granulocytes.

## References

[1] Alessio-Mazzola M Lovisolo S Sonzogni B , et al. Clinical outcome and risk factor predictive for failure of autologous PRP injections for low-to-moderate knee osteoarthritis. J Orthop Surg (Hong Kong). 2021;29(2):23094990211021922. 34180298 10.1177/23094990211021922

[2] Altamura SA Di Martino A Andriolo L , et al. Platelet-rich plasma for sport-active patients with knee osteoarthritis: limited return to sport. Biomed Res Int. 2020;2020:8243865. 10.1155/2020/8243865 PMC7013341 32076616

[3] Bansal H Leon J Pont JL , et al. Platelet-rich plasma (PRP) in osteoarthritis (OA) knee: correct dose critical for long term clinical efficacy. Sci Rep. 2021;11(1):1–10. 33597586 10.1038/s41598-021-83025-2 PMC7889864

[4] Bennell KL Paterson KL Metcalf BR , et al. Effect of intra-articular platelet-rich plasma vs placebo injection on pain and medial tibial cartilage volume in patients with knee osteoarthritis: the RESTORE randomized clinical trial. JAMA. 2021;326(20):2021–2030. 34812863 10.1001/jama.2021.19415 PMC8611484

[5] Biino G Santimone I Minelli C , et al. Age- and sex-related variations in platelet count in Italy: a proposal of reference ranges based on 40987 subjects’ data. PLoS One. 2013;8(1):1–7. 10.1371/journal.pone.0054289 PMC3561305 23382888

[6] Boffa A Andriolo L Franceschini M , et al. Minimal clinically important difference and patient acceptable symptom state in patients with knee osteoarthritis treated with PRP injection. Orthop J Sports Med. 2021;9(10):23259671211026242. 34631901 10.1177/23259671211026242 PMC8495529

[7] Castillo TN Pouliot MA Kim Hyeon Joo Dragoo JL . Comparison of growth factor and platelet concentration from commercial platelet-rich plasma separation systems. Am J Sports Med. 2011;39(2):266–271. 21051428 10.1177/0363546510387517

[8] Centers for Medicare & Medicaid Services (CMS). Billing and coding: platelet rich plasma. Accessed January 18, 2023. https://www.cms.gov/medicare-coverage-database/view/article.aspx?articleid=58351

[9] Cerza F Carnì S Carcangiu A , et al. Comparison between hyaluronic acid and platelet-rich plasma, intra-articular infiltration in the treatment of gonarthrosis. Am J Sports Med. 2012;40(12):2822–2827. 23104611 10.1177/0363546512461902

[10] Di Martino A di Matteo B Papio T , et al. Platelet-rich plasma versus hyaluronic acid injections for the treatment of knee osteoarthritis: results at 5 years of a double-blind, randomized controlled trial. Am J Sports Med. 2019;47(2):347–354. 30545242 10.1177/0363546518814532

[11] Filardo G Previtali D Napoli F Candrian C Zaffagnini S Grassi A . PRP injections for the treatment of knee osteoarthritis: a meta-analysis of randomized controlled trials. Cartilage. 2021;13(1):364S–375S. 32551947 10.1177/1947603520931170 PMC8808870

[12] Global Burden of Disease Collaborative Network. Global Burden of Disease Study 2019. Results. Accessed November 16, 2023. http://ghdx.healthdata.org/gbd-results-tool

[13] Hermans J Bierma-Zeinstra SMA Bos PK Verhaar JAN Reijman M . The most accurate approach for intra-articular needle placement in the knee joint: a systematic review. Semin Arthritis Rheum. 2011;41(2):106–115. 22036252 10.1016/j.semarthrit.2011.02.007

[14] Jones CI . Platelet function and ageing. Mammalian Genome. 2016;27(7-8):358–366. 27068925 10.1007/s00335-016-9629-8 PMC4935731

[15] Kon E Di Matteo B Delgado D , et al. Platelet-rich plasma for the treatment of knee osteoarthritis: an expert opinion and proposal for a novel classification and coding system. Expert Opin Biol Ther. 2020;20(12):1447–1460. 32692595 10.1080/14712598.2020.1798925

[16] Korpershoek JV Vonk LA De Windt TS , et al. Intra-articular injection with autologous conditioned plasma does not lead to a clinically relevant improvement of knee osteoarthritis: a prospective case series of 140 patients with 1-year follow-up. Acta Orthop. 2020;91(6):743–749. 32698659 10.1080/17453674.2020.1795366 PMC8023954

[17] Mazzocca AD Mccarthy MBR Chowaniec DM , et al. Platelet-rich plasma differs according to preparation method and human variability. J Bone Joint Surg. 2012;94(4):308–316. 22336969 10.2106/JBJS.K.00430

[18] Mussano F Genova T Munaron L Petrillo S Erovigni F Carossa S . Cytokine, chemokine, and growth factor profile of platelet-rich plasma. Platelets. 2016;27(5):467–471. 26950533 10.3109/09537104.2016.1143922

[19] Paterson KL Nicholls M Bennell KL Bates D . Intra-articular injection of photo-activated platelet-rich plasma in patients with knee osteoarthritis: a double-blind, randomized controlled pilot study. BMC Musculoskelet Disord. 2016;17:67. 26861957 10.1186/s12891-016-0920-3 PMC4748460

[20] Previtali D Merli G Di Laura Frattura G Candrian C Zaffagnini S Filardo G . The long-lasting effects of “placebo injections” in knee osteoarthritis: a meta-analysis. Cartilage. 2021;13(1S):185S–196S. 10.1177/1947603520906597 PMC8808779 32186401

[21] Riddle DL Stratford PW . Unilateral vs bilateral symptomatic knee osteoarthritis: associations between pain intensity and function. Rheumatology. 2013;52(12):2229–2237. 24026250 10.1093/rheumatology/ket291 PMC3828512

[22] Roos E . KOOS FAQs. Accessed January 25, 2022. http://www.koos.nu/koosfaq.html

[23] Saita Y Kobayashi Y Nishio H , et al. Predictors of effectiveness of platelet-rich plasma therapy for knee osteoarthritis: a retrospective cohort study. J Clin Med. 2021;10(19):4514. 34640529 10.3390/jcm10194514 PMC8509123

[24] Smith PA . Intra-articular autologous conditioned plasma injections provide safe and efficacious treatment for knee osteoarthritis. Am J Sports Med. 2015;44(4):884–891. 10.1177/0363546515624678 26831629

[25] Sun SF Hsu CW Lin HS , et al. A single intraarticular platelet-rich plasma improves pain and function for patients with early knee osteoarthritis: analyses by radiographic severity and age. J Back Musculoskelet Rehabil. 2022;35(1):93–102. 34092592 10.3233/BMR-200193

[26] Von Elm E Altman DG Egger M Pocock SJ Gøtzsche PC Vandenbrouckef JP . The Strengthening the Reporting of Observational Studies in Epidemiology (STROBE) statement: guidelines for reporting observational studies. Bull World Health Organ. 2007;85(11):867–872. 18038077 10.2471/BLT.07.045120 PMC2636253

[27] White D Zhang Y Felson D , et al. The independent effect of pain in one versus two knees on the presence of low physical function: the MOST Study. Arthritis Care Res. 2010;62(7):938–943. 10.1002/acr.20166 PMC2902715 20191572

[28] Zahir H Dehghani B Yuan X , et al. In vitro responses to platelet-rich-plasma are associated with variable clinical outcomes in patients with knee osteoarthritis. Sci Rep. 2021;11(1):1–13. 34075069 10.1038/s41598-021-90174-x PMC8169703

[29] Zhao L Hu M Xiao Q , et al. Efficacy and safety of platelet-rich plasma in melasma: a systematic review and meta-analysis. Dermatol Ther. 2021;11(5):1587–1597. 10.1007/s13555-021-00575-z PMC8484406 34269967

